# Longitudinal analysis of serum oxylipin profile as a novel descriptor of the inflammatory response to surgery

**DOI:** 10.1186/s12967-017-1171-2

**Published:** 2017-04-26

**Authors:** Arnaud M. Wolfer, Alasdair J. Scott, Claudia Rueb, Mathieu Gaudin, Ara Darzi, Jeremy K. Nicholson, Elaine Holmes, James M. Kinross

**Affiliations:** 1 Division of Computational and Systems Medicine, Department of Surgery and Cancer, Imperial College London, London, UK; 2 Imperial College Healthcare NHS Trust, London, UK; 3 Division of Computational and Systems Medicine, Faculty of Medicine, Imperial College London, 6th Floor, Alexander Fleming Building, South Kensington, London, SW7 2AZ UK

**Keywords:** Colorectal surgery, Mass spectrometry, Metabonomics, Oxylipin, Surgery, Systemic inflammatory response syndrome

## Abstract

**Background:**

Oxylipins are potent lipid mediators demonstrated to initiate and regulate inflammation yet little is known regarding their involvement in the response to surgical trauma. As key modulators of the inflammatory response, oxylipins have the potential to provide novel insights into the physiological response to surgery and the pathophysiology of post-operative complications. We aimed to investigate the effects of major surgery on longitudinal oxylipin profile.

**Methods:**

Adults patients undergoing elective laparoscopic or open colorectal resections were included. Primary outcomes were serum oxylipin profile quantified by ultra high-performance liquid chromatography-mass spectrometry, serum white cell count and C-reactive protein concentration. Serum samples were taken at three time-points: pre-operative (day zero), early post-operative (day one) and late post-operative (day four/five).

**Results:**

Some 55 patients were included, of which 33 (60%) underwent surgery that was completed laparoscopically. Pre-operative oxylipin profiles were characterised by marked heterogeneity but surgery induced a common shift resulting in more homogeneity at the early post-operative time-point. By the late post-operative phase, oxylipin profiles were again highly variable. This evolution was driven by time-dependent changes in specific oxylipins. Notably, the levels of several oxylipins with anti-inflammatory properties (15-HETE and four regioisomers of DHET) were reduced at the early post-operative point before returning to baseline by the late post-operative period. In addition, levels of the pro-inflammatory 11-HETE rose in the early post-operative phase while levels of anti-thrombotic mediators (9-HODE and 13-HODE) fell; concentrations of all three oxylipins then remained fairly static from early to late post-operative phases. Compared to those undergoing laparoscopic surgery, patients undergoing open surgery had lower levels of some anti-inflammatory oxylipins (8,9-DHET and 17-HDoHE) in addition to reduced concentrations of anti-thrombotic mediators (9-HODE and 13-HODE) with increased concentration of their pro-thrombotic counterpart (TxB2).

**Conclusions:**

Serum oxylipin profile is modified by surgical intervention and may even be sensitive to the degree of surgical trauma and therefore represents a novel descriptor of the surgical systemic inflammatory response.

**Electronic supplementary material:**

The online version of this article (doi:10.1186/s12967-017-1171-2) contains supplementary material, which is available to authorized users.

## Background

Complications following colorectal surgery, such as surgical site infection (2–25% [1, 2]) and anastomotic leak (3–15% [3]), frequently induce the systemic inflammatory response syndrome (SIRS) or sepsis which are the major causes of post-operative morbidity and mortality [3–6]. To detect such complications, clinicians are generally reliant on the longitudinal assessment of physiological parameters (e.g. respiratory rate, heart rate, temperature) and biomarkers of inflammation, such as C-reactive protein (CRP) and white cell count (WCC). However, pertubations in these parameters are the final common pathway of many distinct pathologies and are therefore relatively late indicators and are extremely non-specific. A more detailed understanding of the inflammatory response and the ability to quantify it with higher resolution may enhance the early detection of acute post-operative complications.

Eicosanoids, and more generally oxylipins, are potent, locally-acting lipid mediators derived from the metabolism of polyunsaturated fatty acid (PUFA) precursors such as linoleic acid (C18:2, LA), dihomo-γ-linolenic acid (C20:3, DGLA), arachidonic acid (C20:4, AA), eicosapentaenoic acid (C20:5, EPA) and docosahexaenoic acid (C22:6, DHA). These PUFAs are principally converted to biologically active downstream mediators by three enzymatic pathways. Cyclooxygenase (COX) produces prostaglandins (PG) and thromboxanes (Tx), while lipoxygenase (LOX) produces leukotrienes (LT). Finally, cytochrome P450 (CYP450), together with LOX, is responsible for generating the less well-characterised “non-classical” eicosanoids including AA-derived hydroxyeicosatetraenoic acids (HETEs) and epoxyeicosatrienoic acids (EETs), LA-derived hydroxyoctadecadienoic acids (HODEs) and DHA-derived hydroxy-docosahexaenoic acids (HDoHEs). Oxylipins form a complex of interlinked cascades with a variety of precursors and metabolites that may or may not have biological activity. The biological effects of oxylipins are diverse, pleiotropic, antagonistic and, in some cases, still unclear. However, their ability to regulate the propagation and resolution of inflammation via effects on smooth muscle, cytokine release, the coagulation cascade and leukocyte chemotaxis is well-described [7–9]. Despite the acknowledged importance of oxylipins, there remains an incomplete knowledge of their systemic profiles during acute inflammation in humans due to a lack, until recently, of suitable quantitative bioanalytical platforms.

We have previously described a novel, highly sensitive assay for the detection in biofluids of a panel of oxylipins and PUFAs spanning the major PUFA precursors and the three principal biosynthetic enzyme pathways (CYP450, COX and LOX) [10]. In the present study, we applied this assay to longitudinally quantify the serum levels of key oxylipins in patients undergoing colorectal surgery. The aims of our investigation were, (1) to provide proof-of-principle that multiple oxylipin concentrations can be quantified simultaneously in human biofluids in the clinical environment and; (2) to better characterise the evolution of the oxylipin profile in response to surgical trauma. We hypothesise that the serum oxylipin profile may provide novel insights into the inflammatory response to surgery and perhaps ultimately show potential as an early biomarker of inflammatory dysregulation.

## Methods

### Patients and samples

This longitudinal observational investigation was conducted at St Mary’s Hospital in London, UK, from January 2012 to April 2013. All patients over the age of 18 undergoing laparoscopic or open elective colorectal resections for both benign and malignant conditions were prospectively recruited to the study by a research nurse on the morning of surgery. Operative approach (e.g. open or laparoscopic) was at the discretion of the operating surgeon and patient and there were no absolute criteria for either. Patients undergoing emergency surgery and those unable to give informed consent were excluded. All patients received standard prophylactic antibiotic treatment on induction of general anaesthesia and were managed post-operatively according to a standardised enhanced recovery program [11].

Primary outcome was the biochemical and inflammatory response to surgery measured by the serum oxylipin profile, white cell count and C-reactive protein concentration. Secondary outcomes included the development of post-operative complications and length of stay. Post-operative complications included anastomotic leak (proven on imaging), severe sepsis (defined according to guidelines from the Surviving Sepsis Campaign [12]) and death. Blood was sampled into serum separator Vacutainers (Becton–Dickinson, Oxford, UK) pre-operatively and daily after surgery until 14 days post-operatively or discharge (whichever came sooner). Blood was allowed to clot in the Vacutainer before being centrifuged at 1200*g* for 10 min to obtain serum that was then frozen (−80 °C) until analysis.

### Mass spectrometric quantification of oxylipins

Sample preparation and the quantification of lipid mediators by ultra high-performance liquid chromatography (UPLC)-mass spectrometry followed the previously published method of Wolfer et al. [10], resulting in the quantification of 21 oxylipins and PUFAs (Table 1). The procedure is described in detail in the Additional file 1 and summarised below. Sample preparation consisted of a solid phase extraction (Oasis MAX µElution, Waters, Milford, USA) of 100 µL serum spiked with deuterated internal standards (Cayman Chemical, MI, USA) to account for variation in extraction yields and instrumentation. UPLC enabled the separation of all oxylipins and PUFAs in 13 min with a HSS T3 UPLC (100*1 mm, 1.8 μm) column maintained at 40 °C on an Acquity UPLC and samples maintained at 4 °C. The mobile phases consisted of H_2_O + 0.1% formic acid (A) and acetonitrile + 0.1% formic acid (B), with a flow rate of 0.14 mL/min. Negative ionisation mode multiple reaction monitoring mass spectrometry enabled the selection of the most specific and sensitive ion for the quantification of each analyte (Additional file 1: Figures S1, S2) using a Xevo TQ-S triple quadrupole mass spectrometer (Waters, Milford, USA). For each batch of 96 samples, a calibration curve was prepared by serial dilution of a mixture of oxylipin (1 ng/μL to 0.01 pg/μL) and PUFA (10 ng/μL to 0.1 pg/μL) standards (Caymen Chemical) in MeOH/H_2_O 1:1 and sample analyte concentrations were calculated from the standard curve. Peak detection, integration and quantification were achieved using the Masslynx and TargetLynx software (Waters, Manchester, UK) and further manual verifications.

**Table 1:** Summary of quantified oxylipins and PUFAs including their metabolism and principal biological activity

Compound	Synonym	PUFA precursor	Enzymatic Pathway	Intrinsic activity	Principal biological action
C20:3 (DGLA)	Dihomo-γ-linolenic acid			Inactive	
C20:4 (AA)	Arachidonic acid			Inactive	
C20:5 (EPA)	Eicosapentaenoic acid			Inactive	
C22:6 (DHA)	Docosahexaenoic acid			Inactive	
9-HODE		LA	LOX (12-LOX)	Active	Increases fibrinolysis
13-HODE		LA	LOX (15-LOX)	Active	Reduces platelets adhesivity
5-HETE		AA	LOX (5-LOX)	Active	Promotes neutrophil degranulation, stimulates calcium mobilisation
8-HETE		AA	LOX (15-LOX)	Active	Inhibits leukocyte migration and promotes wound healing by PPARα activation
11-HETE		AA	COX	Active	Chemo-attractant, promotes neutrophil recruitment
12-HETE		AA	LOX (12-LOX)	Active	Chemo-attractant, promotes neutrophil recruitment
15-HETE		AA	LOX (15-LOX)	Active	Inhibits both neutrophil migration through the endothelium and LTB4 synthesis, activates PPARβ/δ
5,6-DHET		AA	CYP450	Inactive	Stable marker for 5,6-EET which promotes angiogenesis
8,9-DHET		AA	CYP450	Inactive	Stable marker for 8,9-EET which promotes angiogenesis
11,12-DHET		AA	CYP450	Inactive	Stable marker for 11,12-EET which promotes angiogenesis and new tissue formation
14,15-DHET		AA	CYP450	Inactive	Stable marker for 14,15-EET which promotes new tissue formation
14-HDoHE		DHA	LOX (12-LOX)	Active	Promotes wound closure and healing
17-HDoHE		DHA	LOX (15-LOX)	Active	Inhibits pro-inflammatory mechanisms
12-Oxo-LTB4	12-Oxo leukotriene B4	AA	LOX (5-LOX)	Active	Stable marker for LTB4 which is chemo-attractant and promotes neutrophil recruitment
6-Keto-PGF1α	6-Keto prostaglandin F1α	AA	COX	Inactive	Stable marker for PGI2 which inhibits platelet aggregation and induces vasodilatation
PGF2α	Prostaglandin F2α	AA	COX	Active	Promotes vasoconstriction
TxB2	Thromboxane B2	AA	COX	Inactive	Increases platelet aggregation, stimulates activation of new platelets

*HODE* hydroxyoctadecadienoic acid, *HETE* hydroxy eicosatetraenoic acid, *DHET* dihydroxyeicosatrienoic acid, *HDoHE* hydroxy docosahexaenoic acid, *LA* linoleic acid, *EET* epoxyeicosatrienoic acid, *LT* leukotriene, *LOX* leukotriene oxidase, *CYP450* cytochrome P450, *COX* cyclo-oxygenase, *PG* prostaglandin, *PPAR* peroxisome proliferator-activated receptor. Some oxylipins can present a time-dependent inflammatory activity which may be different from the main reported one

### Statistical analysis

Univariate analysis was performed in R v3.1.3 (Foundation for Statistical Computing, Vienna, Austria) [13]. Patients’ demographic and clinical characteristics are reported as median (interquartile range [IQR]) and statistical comparisons were by Mann–Whitney U or Fisher’s exact tests as appropriate. Changes in oxylipin concentration between time-points are reported as median fold change (IQR) and assessed by paired t-tests with application of the Benjamini-Hochberg false discovery rate. Oxylipin concentrations at each time-point were paired with WCC and CRP measurements taken on the same day. WCC and CRP concentrations and associated heat-maps were generated using in-house scripts and plotted using the ggplot2 package [14]. Significance testing of correlation coefficients was achieved by t tests.

Multivariate analysis was performed in R v3.1.3 and SIMCA-P v13 (Umetrics AB, Umeå Sweden). Prior to analysis, analyte concentrations were mean-centred and scaled to unit-variance. Unsupervised (principal component analysis [PCA]) and supervised (partial least squares discriminant analysis [PLS-DA]) methods were employed to visualise and interpret the oxylipin profile in the context of clinical variables including time-point and surgical approach. Goodness of fit of multivariate models was evaluated by the proportion of variance explained by the model (R^2^X) and the predicative ability was estimated by cross-validation (Q^2^Y) and permutation testing (1000 permutations, p value reported).

## Results

### Patients and samples

Some 55 patients were recruited during the 16 month study period—30 females and 25 males. Patient demographics and clinical data are presented in Table 2. During the early post-operative period (up to 30 days or discharge from hospital) a total of five patients (9%) suffered an anastomotic leak and four (7%) developed severe sepsis (three following an anastomotic leak and one with strangulated small bowel in an incisional hernia). One patient (~2%) died.

**Table 2:** Demographic, clinical and surgical characteristics of the recruited population

Clinical variable	Total (*n* = 55)	Open (*n* = 22)	Laparoscopic (*n* = 33)	*p* value
Demographics				
Female	25 (45.5%)	5 (23%)	20 (61%)	0.01287
Age	65 (59–73)	66 (62–76)	65 (59–71)	ns
BMI	25.8 (6.0)	28.5 (25–32)	25 (23–27)	0.01281
Reason for resection				ns
Cancer	49 (89%)	18 (82%)	31 (94%)	
Inflammatory bowel disease	5 (9%)	4 (18%)	1 (3%)	
Diverticular disease	1 (2%)	0	1 (3%)	
Resection				ns
Anterior resection of rectum	22 (40%)	11 (50%)	11 (33%)	
Right hemi colectomy	22 (40%)	6 (27%)	16 (49%)	
Total colectomy	5 (9%)	2 (9%)	3 (9%)	
Left hemi colectomy	3 (5.5%)	0	3 (9%)	
Abdomino-perineal excision of rectum	2 (3.5%)	2 (9%)	0	
Small bowel	1 (2%)	1 (5%)	0	
Secondary outcomes				
Length of stay	8 (6.8)	10 (8–15)	7 (6–10)	0.04692
Severe sepsis	4 (7%)	2 (9%)	2 (6%)	ns
ITU admission	5 (9%)	3 (14%)	2 (6%)	ns
Anastomotic leak	5 (9%)	2 (2%)	3 (9%)	ns
In-hospital death	2 (3.5%)	1 (4.5%)	0	ns

Data are reported as median (IQR) or absolute count (proportion of total) and statistical comparisons were by Mann–Whitney U tests and Fisher’s exact tests respectively. Severe sepsis was defined according to guidelines from the Surviving Sepsis Campaign [12]

Table 2 provides a comparison of demographic variables between patients undergoing open (n = 22, including conversions) versus laparoscopic surgery. Patients in the laparoscopic cohort had a significantly lower BMI (25 [23–27] vs. 28.5 [25–32], *p* = 0.013) than patients undergoing open surgery and were more likely to be female (61 vs. 23%, *p* = 0.013). There were no differences in the procedures, underlying pathologies or development of post-operative complications between the two groups. However, patients undergoing laparoscopic surgery had a significantly shorter length of stay (7 [6–10] vs. 10 [8–15], *p* = 0.047).

To simplify our analysis of the evolving oxylipin profile over time we grouped samples into three defined time-points: pre-operative (day zero), early post-operative (day one) and late post-operative (day four or five, depending on sample availability). A total of 31/55 patients provided a pre-operative sample, 41/55 provided an early post-operative sample and 29/55 provided a late post-operative sample.

### Surgery induces time-dependent shifts in serum oxylipin profiles

**Fig. 1:**
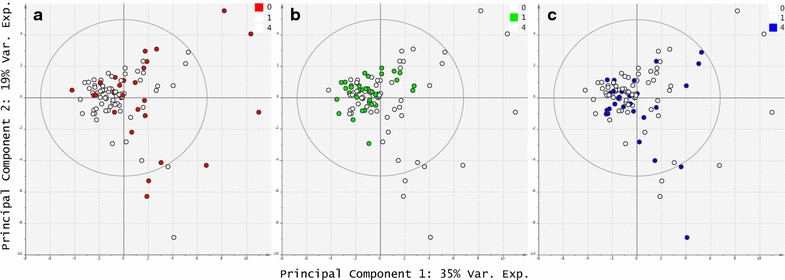
Principal component analysis scores plots of pre- and post-operative oxylipin profiles, highlighting: **a** significant pre-operative variability (pre-operative day, *red*); **b** the common deviation induced by surgery at the early post-operative time-point (day one, *green*) and; **c** renewed heterogeneity by the late post-operative phase (day four, *blue*)

Pre-operatively (Fig. 1a), patients are widely dispersed across the PCA scores plot of their serum oxylipin profiles suggesting significant baseline heterogeneity. In contrast, in the early post-operative phase (day one, Fig. 1b), patients form a tight cluster, implying that surgery induces a common shift in oxylipin profile, which overwhelms the baseline heterogeneity. By the late post-operative time-point (day four, Fig. 1c), patients are again more widely dispersed in the scores plot, regaining significant variability in their oxylipin profiles. Notably, nearly all the outliers from the three time-points (defined by Hotelling’s T2 ellipse) are from the pre-operative phase. These data suggest that although patients may have very different resting oxylipin profiles, they exhibit a similar deviation in response to surgical trauma. However, this homogenous response quickly gives way to renewed heterogeneity as patients follow different inflammatory trajectories during their recovery.

We also constructed partial least squares discriminant analysis (PLS-DA) models to determine if oxylipin profiles alone could be used to differentiate between operative time-points. PLS-DA models demonstrated moderate ability to distinguish between pre-operative and early post-operative samples based on the variance explained by the model (R^2^X = 0.37) and the ability of the model to predict the timepoint (Q^2^Y = 0.24, permutation *p* < 0.001). PLS-DA models comparing early and late post-operative samples (R^2^X = 0.28, Q^2^Y = 0.22, permutation *p* < 0.001) and between pre-operative and late post-operative samples (R^2^X = 0.32, Q^2^Y = 0.39, permutation *p* < 0.001) were also statistically significant.

### Surgery induces time-dependent concentration changes in specific oxylipins

To quantify how the concentrations of specific oxylipins changed over time in response to surgery we compared paired (i.e. from the same patient) oxylipin concentrations between pre-operative and early post-operative time-points, early and late post-operative time-points and pre-operative and late-post-operative time-points. CRP concentration and WCC were similarly compared between time-points to provide a known reference of inflammation. Results are described in Table 3 as median fold changes (IQR) for each metabolite between the specified time-points. As expected, compared to pre-operative samples, both WCC (1.44-fold, *p* < 0.0001) and CRP (30.00-fold, *p* = 0.007) were significantly elevated at the early post-operative point. The pro-inflammatory mediator 11-HETE was also elevated (1.47-fold, *p* = 0.005). At the same time, there was a significant decreases in the concentration of a number of oxylipins reported to have anti-inflammatory effects including 15-HETE (0.63-fold, *p* = 0.005), all four regioisomers of dihydroxyeicosatrienoic acid (DHET) and a trend to reduction in the concentration of 8-HETE (0.74-fold, *p* = 0.052). We also noted reductions in the levels of two lipid mediators with anti-thrombotic effects, 9-HODE (0.71-fold, *p* = 0.028) and 13-HODE (0.72-fold, *p* = 0.039).

**Table 3:** Changes in the concentrations of specific oxylipins, CRP and WCC between time-points

Analyte	Pre- vs. early		Early vs. late		Pre- vs. late	
	Fold change	p value	Fold change	p value	Fold change	p value
CRP	*30.00 (3.26–36.47)*	*0.007*	1.07 (0.74–2.42)	0.213	*13.25 (4.95–34.89)*	*0.0002*
WCC	*1.44 (1.25–1.64)*	*<0.0001*	*0.78 (0.68–0.86)*	*0.045*	1.15 (0.85–1.47)	0.13
9-HODE	*0.71 (0.55–0.90)*	*0.028*	1.01 (0.71–1.46)	0.295	0.71 (0.42–1.48)	0.891
13-HODE	*0.72 (0.61–1.10)*	*0.039*	1.06 (0.70–1.60)	0.117	0.73 (0.52–1.47)	0.955
5-HETE	0.89 (0.70–1.22)	0.138	1.13 (0.80–1.62)	0.18	1.04 (0.72–1.71)	0.445
8-HETE	0.74 (0.25–1.18)	0.052	*1.54 (1.01–2.54)*	*0.011*	1.28 (0.96–1.72)	0.426
12-HETE	0.86 (0.41–1.74)	0.214	1.62 (0.64–4.46)	0.065	2.57 (0.66–5.95)	0.467
12-Oxo-LTB4	0.53 (0.28–1.05)	0.147	1.00 (0.49–3.16)	0.118	*0.51 (0.31–1.00)*	*0.039*
8,9-DHET	*0.62 (0.34–0.88)*	*0.024*	*1.35 (0.94–3.57)*	*0.014*	0.93 (0.67–1.79)	0.799
5,6-DHET	*0.94 (0.08–1.00)*	*0.035*	1.00 (1.00–14.03)	0.151	1.00 (1.00–1.39)	0.226
11,12-DHET	*0.70 (0.37–0.93)*	*0.016*	*1.23 (0.88–2.06)*	*0.038*	0.87 (0.63–1.28)	0.474
14,15-DHET	*0.66 (0.42–0.98)*	*0.043*	*1.19 (1.08–1.93)*	*0.03*	1.03 (0.62–1.72)	0.665
PGF2α	0.99 (0.29–1.03)	0.311	1.00 (0.19–6.44)	0.377	1.50 (0.41–7.72)	0.46
6-Keto-PGF1α	1.00 (0.64–1.61)	0.914	1.00 (0.41–1.43)	0.127	1.00 (0.44–1.14)	0.468
TXB2	0.51 (0.05–3.67)	0.227	1.21 (0.25–7.56)	0.365	1.70 (0.23–13.32)	0.769
11-HETE	*1.47 (1.18–1.65)*	*0.005*	1.20 (0.89–2.10)	0.068	1.17 (0.60–1.86)	0.756
14-HDoHE	0.96 (0.41–1.76)	0.275	1.26 (0.75–2.56)	0.057	1.51 (0.54–3.01)	0.415
C20:5 (EPA)	0.77 (0.51–1.21)	0.18	1.09 (0.66–1.61)	0.976	0.90 (0.56–1.45)	0.374
C20:4 (AA)	0.92 (0.79–1.29)	0.749	1.14 (0.85–1.50)	0.083	*1.19 (0.90–1.62)*	*0.028*
15(S)-HETE	*0.63 (0.52–0.94)*	*0.005*	1.22 (0.78–1.46)	0.238	0.81 (0.74–1.31)	0.692
C22:6 (DHA)	0.80 (0.58–1.20)	0.352	1.02 (0.78–1.32)	0.948	0.81 (0.66–1.41)	0.675
17-HDoHE	0.82 (0.63–1.26)	0.122	1.19 (0.75–1.75)	0.098	1.16 (0.73–1.27)	0.778
C20:3 (DGLA)	0.96 (0.66–1.27)	0.51	1.00 (0.79–1.50)	0.584	0.98 (0.76–1.34)	0.879

Values are reported as median fold change (interquartile range). Italics indicate statistically significant comparisons

Some of these changes were reversed in the transition from the early to late post-operative time-point. WCC was significantly reduced (0.78-fold, *p* = 0.045) and the concentrations of the anti-inflammatory 8-HETE (1.54-fold, *p* = 0.011) and three of the regioisomers of DHET had all risen towards baseline levels. However, concentrations of 11-HETE showed a trend towards further elevation (1.20-fold, *p* = 0.068), while CRP, 9-HODE and 13-HODE remained fairly static. Levels of the relatively inactive stable degradation product of the pro-inflammatory leukotriene B4, 12-oxo-LTB4, which had demonstrated a trend towards reduction at the early post-operative phase, also remained static and were found to be nearly half their pre-operative values by the late post-operative time-point (0.51-fold, *p* = 0.039).

### Correlation between oxylipins and other known mediators of inflammation over time

**Fig. 2:**
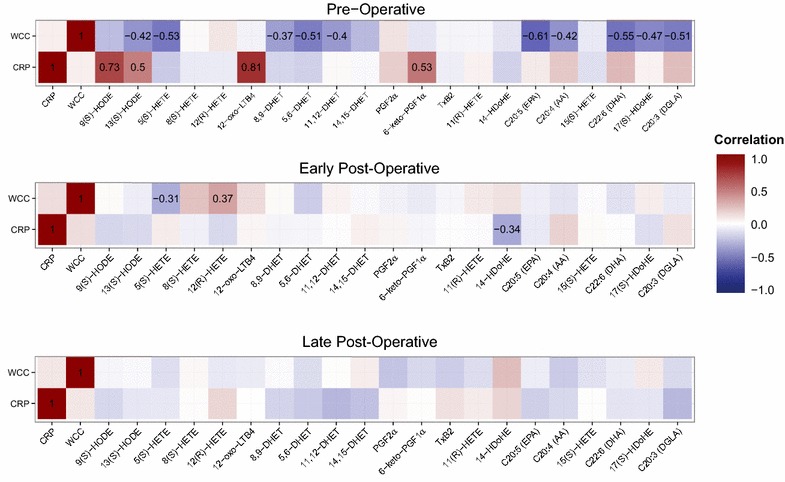
Pearson correlation heat maps between CRP, WCC and oxylipins at the pre-operative, early post-operative and late post-operative time-points. Correlation values are reported for correlations with a p value <0.05 (by *t* test). *Red cells* indicate positive correlation and *blue cells* negative correlation

The relationship between oxylipins and known mediators of inflammation in response to a surgical stimulus is unknown. Therefore, we correlated each mediator with CRP concentration and WCC at each time-point and generated correlation heatmaps of the results (Fig. 2; Additional file 1: Table S1). The majority of statistically significant correlations between WCC, CRP and oxylipins were observed pre-operatively. However, none of these associations persisted consistently at both the early and late post-operative time points. 5-HETE was negatively correlated with WCC pre-operatively (r = −0.53, *p* = 0.0031) and at the early post-operative phase (r = −0.31, *p* = 0.05) but this association was lost by the late post-operative time-point (r = −0.1, *p* = 0.41). In fact, at the late post-operative point, there were no significant correlations between individual oxylipin levels and WCC or CRP concentrations. As expected, we did observe strong positive correlations at all time-points between specific oxylipins and their corresponding PUFA precursors (e.g. 5-HETE and arachidonic acid) and between oxylipins sharing the same biosynthetic cascade (e.g. 8-HETE and 15-HETE synthesised by arachidonate 15-lipoxygenase), which supports the validity of our assay (Additional file 1: Figure S3).

### Post-operative oxylipin profile differs between open and laparoscopic surgery

Open surgery may result in a greater traumatic burden to the patient than laparoscopic surgery [15–21], which might be reflected in differences in their post-operative oxylipin profiles. We therefore generated PLS-DA models to investigate if post-operative WCC, CRP concentration and oxylipin profile could discriminate between open and laparoscopic surgery. A model at the early post-operative time-point (R^2^X = 0.09, Q^2^Y = −0.15, permutation *p* = 0.36) was unable to discriminate reliably but at the late post-operative point, the inflammatory panel demonstrated a modest capacity to predict the operative approach (R^2^Y = 0.28, Q^2^Y = 0.11, permutation *p* < 0.001).

**Fig. 3:**
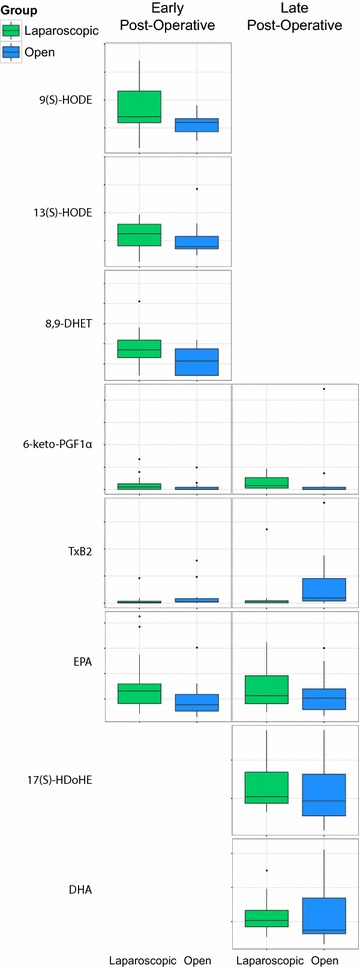
Boxplots of relative concentrations for the oxylipins that differed significantly between laparoscopic (*green*) and open surgery (*blue*) at the early post-operative (*left*) and late post-operative time-points (*right*). All boxplots of concentration (*y-axis*) start at 0 and the scale is preserved for each oxylipin across time and surgery. The *bottom* and *top* of each *box* represent the 1st and 3rd quartiles respectively, while the *centre band* represents the median value [22]. *Whiskers* extend to one standard deviation above and below the mean of the data, while measurements past the whiskers are plotted as possible outliers by a *dot*

We then compared mean oxylipin concentrations between patients undergoing open versus laparoscopic surgery at each time-point (represented by boxplots [22] in Fig. 3). As expected, open and laparoscopic surgery groups displayed no baseline differences in oxylipin concentration at the pre-operative time-point. However, by the early post-operative time-point significant differences between the two groups were noted. Specifically, patients undergoing open surgery had lower levels of 8,9-DHET (on average 29% lower, *p* = 0.047), a stable degradation product of the anti-inflammatory epoxyeicosatrienoic acid and lower concentrations of the anti-thrombotic 9-HODE (on average 31% lower, *p* = 0.0052) and 13-HODE (on average 14% lower, *p* = 0.048) compared to patients undergoing laparoscopic surgery. Furthermore, TxB2 (an inactive metabolite of platelet-activating TxA2) concentration was elevated in the open group (on average 194% higher, *p* = 0.043), while there was a trend towards elevated concentrations of 6-keto-PGF1α (inactive metabolite of PGI_2_, the physiological antagonist of TxA2) in the laparoscopic group (on average 79% higher, *p* = 0.068). Both of these differences persisted into the late post-operative phase, when we also found higher levels of 17-HDoHE (on average 29% higher, *p* = 0.049), a potent anti-inflammatory mediator, and its precursor DHA (on average 51% higher, *p* = 0.05) in patients undergoing laparoscopic surgery. Lastly, concentrations of the PUFA precursor EPA were elevated in the laparoscopic group at both post-operative time-points. There were no significant differences in WCC or CRP concentration between the two surgical approaches at either post-operative time-point.

## Discussion

Oxylipins, such as prostaglandins and leukotrienes, have been individually studied for over fifty years but technological limitations restricting the number of oxylipins that could be simultaneously assayed have necessarily focussed work on allocating distinct regulatory functions to individual mediators [23, 24]. Recent developments in analytical platforms allowed us to develop an assay for the simultaneous analysis of numerous lipid mediators in biofluids [10]. In the present investigation we applied this oxylipin assay to adult patients undergoing elective colorectal surgery to demonstrate that oxylipin profiling is feasible in a clinical context and that it has the potential to provide novel insights into the systemic inflammatory response.

PCA of oxylipin profiles demonstrated that surgery induces profound time-dependent shifts. We observed significant variability amongst pre-operative oxylipin profiles that may reflect the heterogeneity in the demographics (e.g. age, gender, physical condition), underlying aetiologies, attendant comorbidities and pre-operative preparations of the patient population. However, it should be noted that previous studies have shown that even when individuals were well-matched with regard to gender, health and physical condition, their basal oxylipin levels still differed significantly [25]. Despite this underlying heterogeneity, surgery induced a more uniform oxylipin profile at the early post-operative phase, perhaps reflecting a coherent response to surgical trauma. By the late post-operative phase, significant variability in oxylipin profile was again noted, perhaps reflecting unique recovery trajectories between patients. Visual differences between the PCA scores plots were confirmed by supervised multivariate analysis which was able to discriminate between each time-point on the basis of the oxylipin profile.

Univariate analysis of individual oxylipin concentrations revealed that, compared to pre-operative levels, there was a generalised dampening of several anti-inflammatory (15-HETE, 8-HETE and degradation products of EET) and anti-thrombotic (9-HODE and 13-HODE) mediators, combined with an increase in the pro-inflammatory 11-HETE. In the late post-operative phase, the levels of anti-inflammatory mediators were typically elevated compared to the early post-operative time-point suggesting a return towards baseline and indeed, no significant differences were observed for these mediators between the pre-operative and late post-operative samples. In contrast, the pro-inflammatory 11-HETE and anti-thrombotic 9-HODE and 13-HODE did not appear to return to baseline, although the differences between pre-operative and late post-operative measurements were non-significant. Interestingly, levels of 12-oxo-LTB4 (the stable degradation product of pro-inflammatory leukotriene B4) showed a trend towards reduction at the early post-operative point and were significantly reduced by the late post-operative phase. We attempted to correlate oxylipin concentrations with with CRP concentration and WCC as known references of inflammation. However, no consistent correlations we found, perhaps reflecting temporal differences in the dynamics of oxylipin, CRP and WCC levels [26, 27]. When interpreting our results it is important to bear in mind that, due to the lipid mediator’s bioactivity as inflammatory signalling compounds, sample collection and handling could result in in vivo and ex vivo alterations [28]. For example, the stimulation of platelets and leukocytes following venipuncture and serum formation could potentially cause activation of the oxylipin biosynthetic cascade [29]. Equally, anticoagulants used for the collection of plasma (such as heparin) can stimulate oxylipin production via activation of phospholipase A_2_ [30, 31]. However, the practicalities of collecting samples within a clinical environment limit the extent to which such potential effects can be prevented.

Although it is interesting to postulate on how changes in the oxylipin profile fit with the current understanding of the inflammatory response to surgery, we urge caution to avoid overstating and oversimplifying the results of our single study. That said, the early reduction in the concentration of mediators with anti-inflammatory and anti-thrombotic effects could be viewed as “releasing the brake” on inflammation, consistent with the pro-inflammatory, pro-thrombotic phenotype that is thought to dominate the early post- operative phase [32, 33]. Early elevation of pro-inflammatory 11-HETE would also support this hypothesis. The subsequent return of the anti-inflammatory mediators towards basal levels may reflect a reinstatement of normal inflammatory homeostasis. Evidence of early and persistent reduction in the pro-inflammatory leukotriene B4 may seem contrary to this hypothesis but other investigations have demonstrated that leukotriene B4 release by neutrophils peaks extremely rapidly following surgery (e.g. within 6 h) and that levels remain low thereafter [34, 35]. The early post-operative sample in the present study was taken at 24 h, and may well have missed an initial peak in pro-inflammatory oxylipin concentration, leaving only the later nadir. Strassburg et al. recently investigated the effects of on-pump cardiac surgery on serum oxylipin levels in five patients and demonstrated increases in other oxylipins with pro-inflammatory effects at 24 h post-operatively, including 12-HETE and 5-HETE which were quantified in our assay [36]. However, the large physiological insult of on-pump cardiac surgery, small sample size and discrepancies in their sampling protocol (venous blood pre-operatively and arterial blood post-operatively) makes direct comparison between our investigations difficult.

The immunophysiological impact of laparoscopic versus open colorectal surgery is not well understood with conflicting evidence [15–21]. Our investigation was not powered to detect differences in oxylipin profile by outcome (e.g. the development of complications) or by surgical approach as patients were not randomised. With these caveats, we did find that length of stay was shorter in the laparoscopic group and demonstrated interesting differences in the post-operative oxylipin profiles between the two approaches. Patients undergoing open surgery had lower levels of some anti-inflammatory oxylipins (8,9-DHET and 17-HDoHE) in addition to reduced concentrations of anti-thrombotic mediators (9-HODE and 13-HODE) with increased concentration of their pro-thrombotic counterpart (TxB2). Higher levels of anti-inflammatory 17-HDoHE were found amongst the laparoscopic cohort. Whilst almost certainly an oversimplification, these results could be suggestive of a more pro-inflammatory, pro-thrombotic phenotype amongst patients undergoing open surgery.

## Conclusion

This is the first investigation to quantify systemic oxylipin profile longitudinally in a cohort of patients undergoing major surgery. We have demonstrated that surgery induces a common shift in lipid mediators, marked at 24 h post-operatively by a combination of elevation in pro-inflammatory oxylipin concentration with suppression of specific oxylipins thought to have anti-inflammatory and/or anti-thrombotic effects. Furthermore, differences between patients undergoing open versus laparoscopic surgery suggests that the oxylipin profile may be sensitive to the degree of surgical trauma. Based on this evidence, the stage is set to more completely define the “normal” oxylipin dynamics in response to surgery using a more comprehensive sampling strategy and a larger patient cohort. Deviations from this “normal” profile may then offer a useful prognostic tool for the early identification of patients at higher risk of post-operative complications.

## Additional file

**Additional file 1.** Supplementary material containing detailed methodology for mass spectrometric quantification of oxylipins, representative chromatograms and supplementary data.

## Abbreviations

AA: arachidonic acid (C20:4)
BMI: body mass index
COX: cyclooxygenase
CRP: C-reactive protein
CYP450: cytochrome P450
DHA: docosahexaenoic acid (C22:6)
DHET: dihydroxyeicosatrienoic acid
EET: epoxyeicosatrienoic acid
EPA: eicosapentaenoic acid (C20:5)
HDoHE: hydroxydocosahexaenoic acid
HETE: hydroxyeicosatetraenoic acid
HODE: hydroxyoctadecadienoic acid
IQR: inter-quartile range
ITU: intensive treatment unit
LA: linoleic acid (C18:2)
LOX: lipoxygenase
LT: leukotriene
PCA: principal component analysis
PG: prostaglandin
PLS-DA: partial least squares-discriminant analysis
PUFA: polyunsaturated fatty acid
SIRS: systemic inflammatory response syndrome
Tx: thromboxane
UPLC: ultra-high performance liquid chromatography
WCC: white blood cells count
