# Supplementary material for: Longitudinal analysis of serum oxylipin profile as a novel descriptor of the inflammatory response to surgery
Source: J Transl Med. 2017 Apr 26;15:83. doi: 10.1186/s12967-017-1171-2 (PMC5405545; doi:10.1186/s12967-017-1171-2)
Supplement: Supplementary file 1 — Additional file 1. Supplementary material containing detailed methodology for mass spectrometric quantification of oxylipins, representative chromatograms and supplementary data. [file 12967_2017_1171_MOESM1_ESM.doc]

**Longitudinal Analysis of Serum Oxylipin Profile as a Novel Descriptor of the Inflammatory Response to Surgery**

**Arnaud M Wolfer PhD, Alasdair J Scott PhD, Claudia Rueb,** **Mathieu Gaudin PhD, Ara Darzi** KBE HonFrEng FMedSci**, Jeremy K Nicholson** PhD FRCPath FMedSci, **Elaine Holmes PhD, James M Kinross PhD.**

**Supplementary Information**

**Mass Spectrometric Quantification of Oxylipins**

The ultra high-performance liquid chromatography (UPLC)-mass spectrometry assay employed in the present study has been developed to quantify 48 lipid mediators of inflammation in 100μL of serum, plasma or urine, and validated in accordance with the Food and Drug Administration guidelines on Bioanalytical Methods Validation.

Authentic standards were obtained for each analyte and deuterated internal standard. During the method’s development, the most specific and sensitive multiple reaction monitoring transitions were selected to ensure selectivity, avoid interferences (sufficient discrimination from co-eluting oxylipins) and increase detection limits. Oxylipins and their deuterated analogues were also evaluated for crosstalk and carryover and rejected from the panel when results were not satisfactory.

To account for variation in extraction yields and instrument variability, 7 isotopically labelled oxylipins and PUFAs were spiked in the samples before solid phase extraction. When a deuterated analogue was not available, the closest surrogate was selected. Therefore, for quantification purposes, each oxylipin class was assigned a representative deuterated internal standard.

Prior to analysis a standard mixture is prepared by pooling the 48 commercial stock solutions to a final concentration of 2ng/μL for oxylipins and 20ng/μL for PUFAs in MeOH/H2O 1:1. For each batch of 96 samples, corresponding to 24 hours of UHPLC data acquisition, a new calibration is established. Calibration curves are prepared by serial dilution in MeOH/H2O 1:1 of the standard mixture of 48 targeted analytes, ranging from 1ng/μL to 0.01pg/μL for oxylipins (equivalent to 10ng/μL - 0.1pg/μL for PUFAs). Due to the diversity of linear ranges observed across all the targeted analytes, the preparation of a common 13 points calibration curve ensured each oxylipin is covered by the minimum 6 calibration points whilst simplifying the preparation steps. All calibration points are therefore not required or expected for each analyte.

UPLC enabled the separation of 48 oxylipins and PUFAs in 13 min with a HSS T3 UPLC (100*1 mm, 1.8 μm) column maintained at 40°C on an Acquity UPLC, with samples maintained at 4°C. The mobile phases consisted of H_2_O + 0.1% formic acid (A) and acetonitrile + 0.1% formic acid (B), with a flow rate of 0.14 mL/min. The injection volume was 5 μL and all analytes were eluted in a linear gradient from 30 to 70% B between 0 and 12 min; 100% B was held between 12.1 and 13.1 min and the column was reequilibrated to starting conditions between 13.1 and 15 min.

Negative ionisation mode multiple reaction monitoring mass spectrometry enabled the selection of the most specific and sensitive ion for the quantification of each analyte (Figure S1 and S2).

For each analytical batch and each analyte, the lowest limit of quantification (LLOQ), linear range and analyte-response equations are established using the corresponding calibration curve. For each linear standard curve, the ratio of analyte area to its corresponding internal standard area is plotted against nominal concentration, the fit is linear and no weighting factor is applied. Standard concentrations are then back-calculated from the constructed calibration curves. In accordance with FDA guidelines, LLOQ was established as the lowest concentration that results in a peak with a signal-over-noise > 5, satisfying the precision and accuracy criteria during method validation, while also being part of the linear range of the calibration curve (back-calculated residuals <20% for LLOQ, <15% for all other calibration points).

For data analysis purposes, analyte measurements inferior to the LLOQ were replaced by the noise value measured in a blank sample. Finally, as the study data was generated over 3 consecutive batches, run-order effects and batch effects were investigated; no valid statistical models predicting run-order or acquisition batch could be generated, discarding the possibility of such effects.

**Figure S1:** Annotated total ion chromatogram overlaying the 44 multiple reaction monitoring (MRM) functions employed for the targeted quantification of 48 lipid mediators and 7 deuterated IS in an equimolar mixture of commercially available standards.

AA, arachidonic acid; DGLA, dihomo-γ-linolenic acid; DHA, docosahexaenoic acid; DHET, dihydroxyeicosatrienoic acid; DiHDoHE, dihydroxydocosahexaenoic acid; EET, epoxyeicosatrienoic acid; EPA, eicosapentaenoic acid; HDoHE, hydroxydocosahexaenoic acid; HEPE, hydroxypentaenoic acid; HETE, hydroxyeicosatetranoic acid; HODE, hydroxyoctadecadienoic acid; LA, linoleic acid; LT, leukotriene; Lx, Lipoxin; PG, prostaglandin; PUFA, polyunsaturated fatty acids; Rv, Resolvin; Tx, Thromboxane.

**
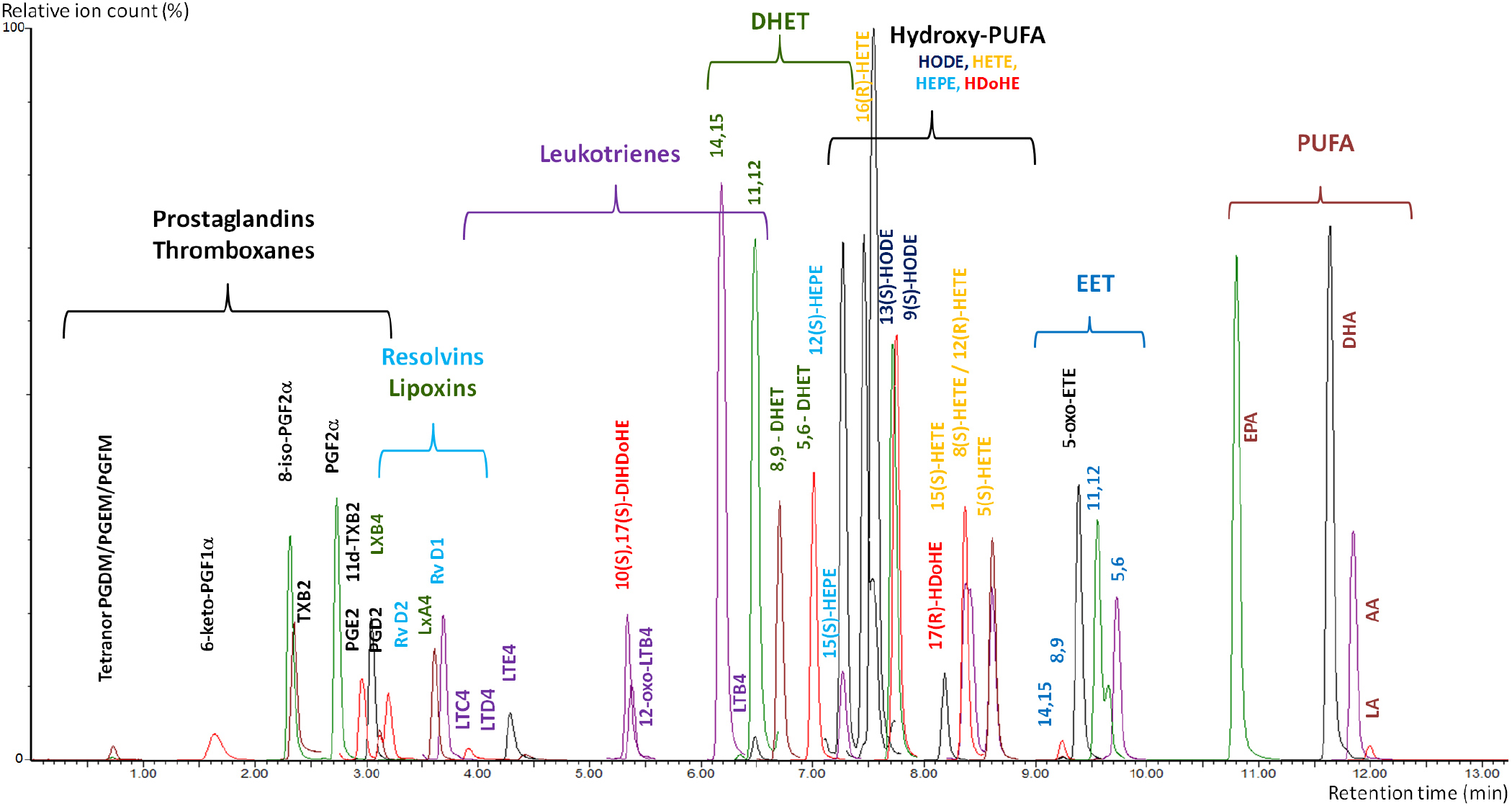
**

**Figure S2:** Total ion chromatogram of a serum sample, focussing on the oxylipin and polyunsaturated fatty acids quantitative transitions. N.B. Due to variation in endogenous levels, not all 48 lipid mediators are visible in every spectrum.

**
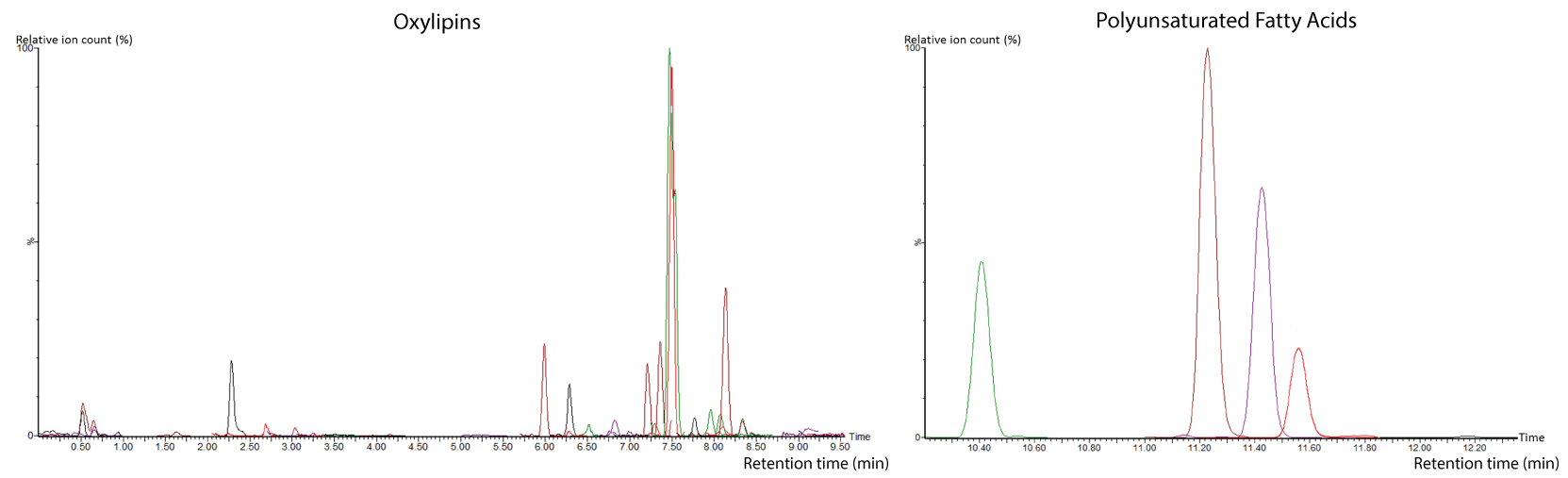
**

**Table S1:** Pearson correlation coefficients and associated *p*-values for correlations between oxylipin concentrations and WCC and CRP level at each time-point. Correlations with significant *p*-values are highlighted.

|  | **Pre-Operative** | | | | **Early Post-Operative** | | | | **Late Post-Operative** | | | |
| --- | --- | --- | --- | --- | --- | --- | --- | --- | --- | --- | --- | --- |
|  | **CRP** | | **WCC** | | **CRP** | | **WCC** | | **CRP** | | **WCC** | |
|  | **r** | **p value** | **r** | **p value** | **r** | **p value** | **r** | **p value** | **r** | **p value** | **r** | **p value** |
| 9(S)-HODE | 0.73 | 0.0015 | -0.32 | 0.0866 | -0.21 | 0.2099 | 0.01 | 0.9316 | -0.19 | 0.3207 | -0.04 | 0.8287 |
| 13(S)-HODE | 0.50 | 0.0479 | -0.42 | 0.0241 | -0.19 | 0.2572 | -0.08 | 0.6213 | -0.12 | 0.5395 | -0.03 | 0.8764 |
| 5(S)-HETE | -0.25 | 0.3413 | -0.53 | 0.0031 | 0.09 | 0.5808 | -0.31 | 0.0479 | -0.11 | 0.5611 | -0.16 | 0.4143 |
| 8(S)-HETE | -0.10 | 0.7075 | 0.03 | 0.8818 | -0.07 | 0.6625 | 0.30 | 0.0627 | 0.01 | 0.9555 | 0.04 | 0.8573 |
| 12(R)-HETE | -0.10 | 0.7262 | 0.12 | 0.5340 | -0.18 | 0.2666 | 0.37 | 0.0205 | 0.21 | 0.2934 | -0.11 | 0.5689 |
| 12-oxo-LTB4 | 0.81 | 0.0001 | -0.06 | 0.7560 | 0.04 | 0.7909 | 0.18 | 0.2670 | 0.00 | 0.9957 | -0.10 | 0.6200 |
| 8,9-DHET | -0.20 | 0.4506 | -0.37 | 0.0453 | -0.05 | 0.7603 | 0.02 | 0.8997 | -0.21 | 0.2834 | -0.02 | 0.9187 |
| 5,6-DHET | -0.23 | 0.3946 | -0.51 | 0.0047 | -0.04 | 0.7887 | -0.24 | 0.1338 | -0.26 | 0.1786 | -0.16 | 0.4016 |
| 11,12-DHET | 0.03 | 0.9181 | -0.40 | 0.0314 | 0.00 | 0.9893 | 0.06 | 0.7171 | -0.35 | 0.0654 | 0.00 | 0.9800 |
| 14,15-DHET | -0.03 | 0.9064 | -0.34 | 0.0705 | 0.07 | 0.6654 | -0.03 | 0.8527 | -0.30 | 0.1164 | 0.08 | 0.6675 |
| PGF2α | 0.24 | 0.3670 | 0.14 | 0.4840 | 0.05 | 0.7501 | -0.07 | 0.6754 | 0.04 | 0.8311 | -0.29 | 0.1361 |
| 6-keto-PGF1α | 0.53 | 0.0345 | -0.13 | 0.5165 | -0.05 | 0.7830 | -0.10 | 0.5399 | 0.01 | 0.9728 | -0.19 | 0.3295 |
| TxB2 | -0.08 | 0.7675 | -0.05 | 0.7884 | -0.01 | 0.9729 | -0.05 | 0.7715 | 0.14 | 0.4696 | -0.24 | 0.2202 |
| 11(R)-HETE | 0.06 | 0.8192 | -0.04 | 0.8230 | -0.02 | 0.9200 | 0.11 | 0.5078 | 0.10 | 0.6285 | -0.16 | 0.4096 |
| 14-HDoHE | -0.22 | 0.4044 | -0.14 | 0.4786 | -0.34 | 0.0335 | 0.14 | 0.3937 | 0.21 | 0.2767 | 0.32 | 0.0998 |
| C20:5 (EPA) | 0.10 | 0.7119 | -0.61 | 0.0005 | -0.10 | 0.5363 | -0.08 | 0.6178 | -0.23 | 0.2369 | -0.09 | 0.6488 |
| C20:4 (AA) | 0.27 | 0.3167 | -0.42 | 0.0222 | 0.22 | 0.1720 | -0.13 | 0.4211 | -0.19 | 0.3293 | -0.25 | 0.1941 |
| 15(S)-HETE | -0.16 | 0.5604 | -0.11 | 0.5784 | 0.01 | 0.9317 | -0.02 | 0.9131 | 0.00 | 0.9824 | -0.15 | 0.4569 |
| C22:6 (DHA) | 0.34 | 0.1925 | -0.55 | 0.0019 | 0.00 | 0.9857 | -0.15 | 0.3600 | -0.13 | 0.5095 | -0.10 | 0.6286 |
| 17(S)-HDoHE | 0.06 | 0.8250 | -0.47 | 0.0107 | -0.16 | 0.3275 | 0.09 | 0.5890 | -0.13 | 0.5252 | 0.11 | 0.5894 |
| C20:3 (DGLA) | 0.32 | 0.2217 | -0.51 | 0.0044 | 0.15 | 0.3680 | -0.10 | 0.5226 | -0.35 | 0.0685 | -0.18 | 0.3592 |

**Figure S3:** Pearson correlation heat maps for the pre-operative (**A**), early post-operative (**B**) and late post-operative (**C**) time-points. Correlations with significant p values (< 0.05 with **Benjamini-Hochberg false discovery rate applied)** are specified.

**
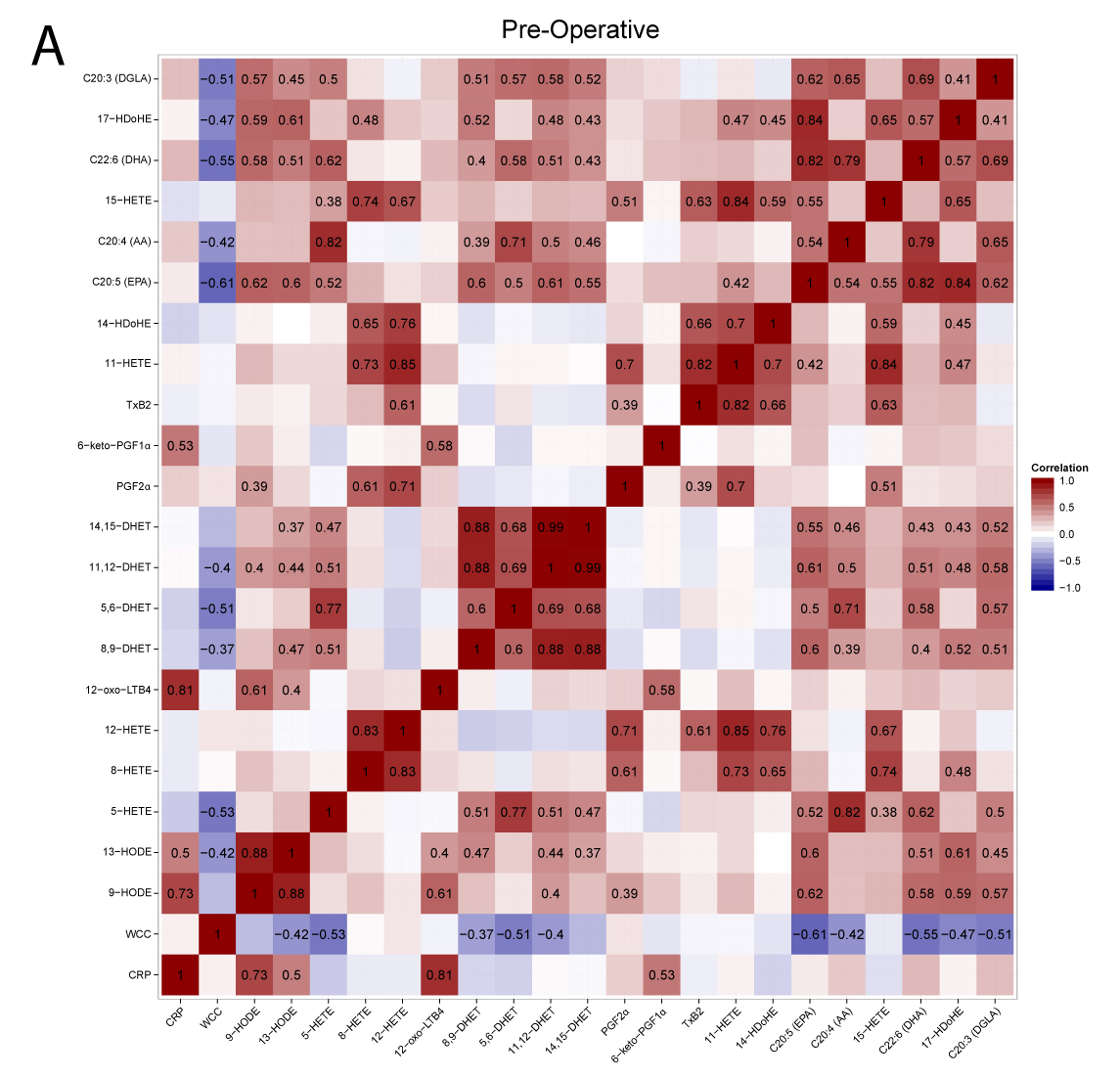
**

**
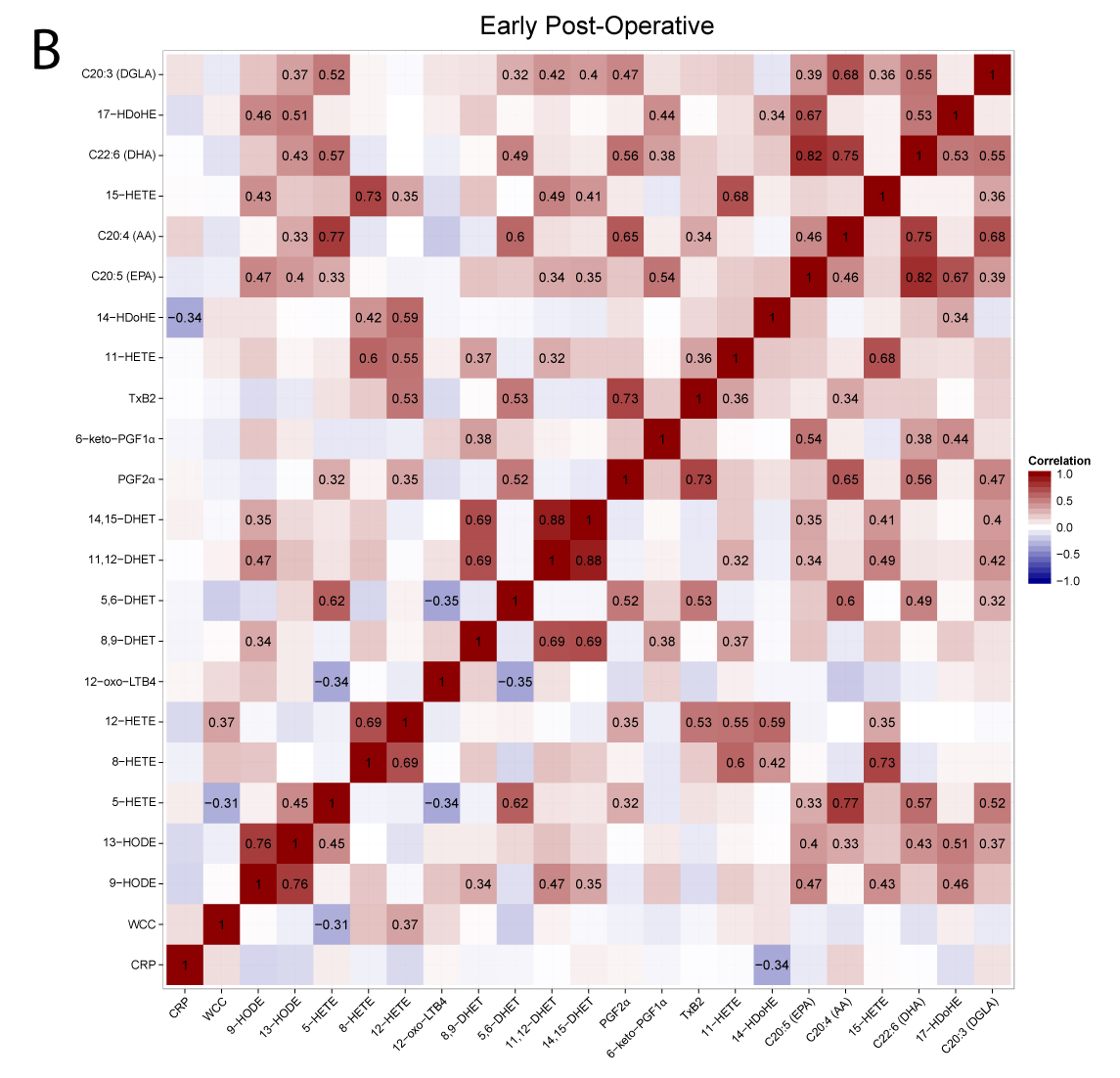
**

**
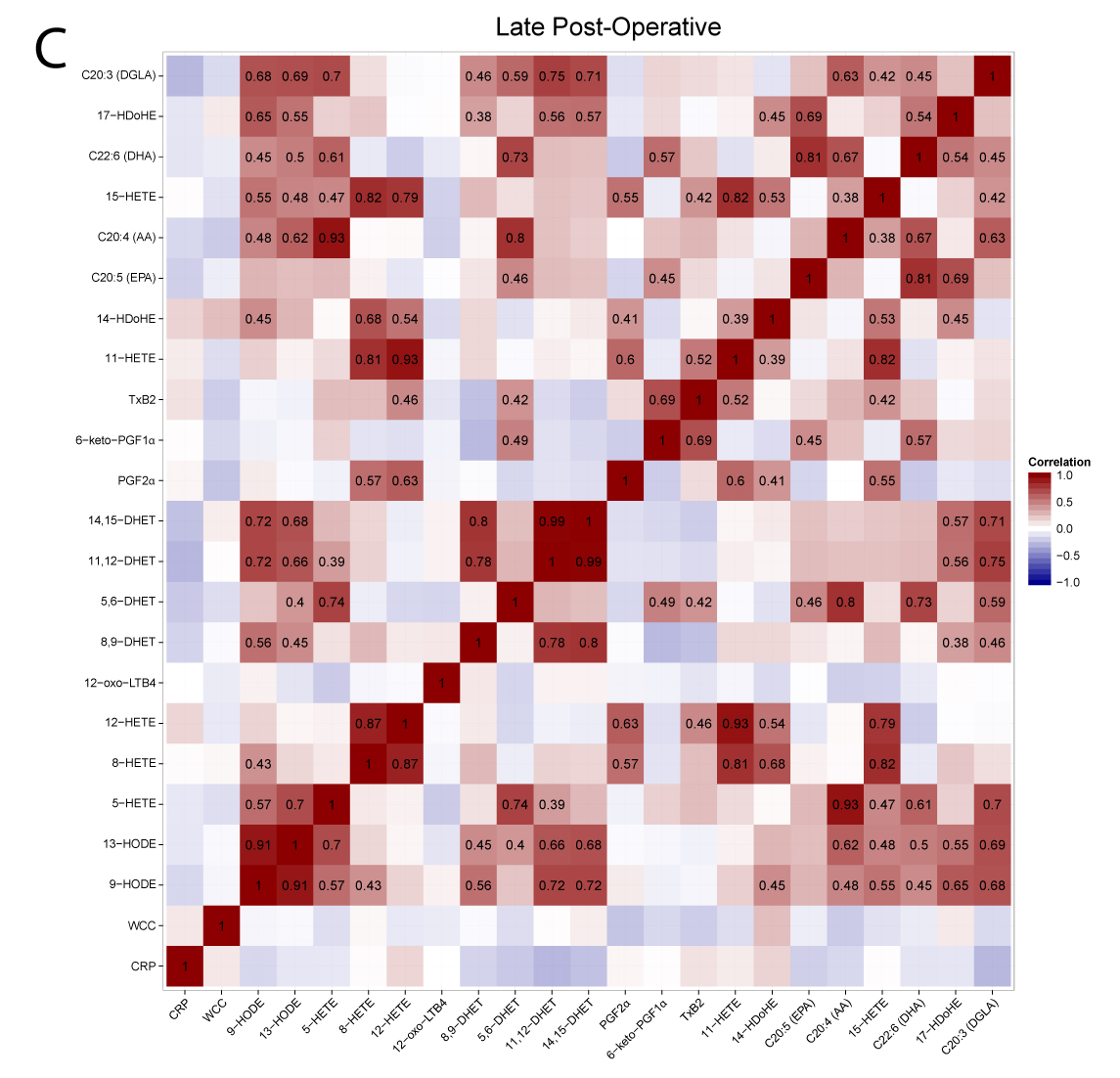
**
